# Human trophoblast-derived exosomes attenuate doxorubicin-induced cardiac injury by regulating miR-200b and downstream Zeb1

**DOI:** 10.1186/s12951-020-00733-z

**Published:** 2020-11-20

**Authors:** Jie Ni, Yihai Liu, Lina Kang, Lian Wang, Zhonglin Han, Kun Wang, Biao Xu, Rong Gu

**Affiliations:** 1 Department of Cardiology, Nanjing Drum Tower Hospital, Clinical College of Nanjing Medical University, Nanjing, 210008 Jiangsu People’s Republic of China; 2 Department of Cardiology, Affiliated Drum Tower Hospital, Medical School of Nanjing University, Nanjing, 210008 Jiangsu People’s Republic of China

**Keywords:** Trophoblasts, Exosomes, miR-200b, Heart failure

## Abstract

Human trophoblast stem cells (TSCs) have been confirmed to play a cardioprotective role in heart failure. However, whether trophoblast stem cell-derived exosomes (TSC-Exos) can protect cardiomyocytes from doxorubicin (Dox)-induced injury remains unclear. In the present study, TSC-Exos were isolated from the supernatants of human trophoblasts using the ultracentrifugation method and characterized by transmission electron microscopy and western blotting. In vitro, primary cardiomyocytes were subjected to Dox and treated with TSC-Exos, miR-200b mimic or miR-200b inhibitor. Cellular apoptosis was observed by flow cytometry and immunoblotting. In vivo, mice were intraperitoneally injected into Dox to establish a heart failure model. Then, different groups of mice were administered either PBS, adeno-associated virus (AAV)-vector, AAV-miR-200b-inhibitor or TSC-Exos via tail vein injection. Then, the cardiac function, cardiac fibrosis and cardiomyocyte apoptosis in each group were evaluated, and the downstream molecular mechanism was explored. TSC-Exos and miR-200b inhibitor both decreased primary cardiomyocyte apoptosis. Similarly, mice receiving TSC-Exos and AAV-miR-200b inhibitor exhibited improved cardiac function, accompanied by reduced apoptosis and inflammation. The bioinformatic prediction and luciferase reporter results confirmed that Zeb1 was a downstream target of miR-200b and had an antiapoptotic effect. TSC-Exos attenuated doxorubicin-induced cardiac injury by playing antiapoptotic and anti-inflammatory roles. The underlying mechanism could be an increase in Zeb1 expression by the inhibition of miR-200b expression. In summary, this study sheds new light on the application of TSC-Exos as a potential therapeutic tool for heart failure.
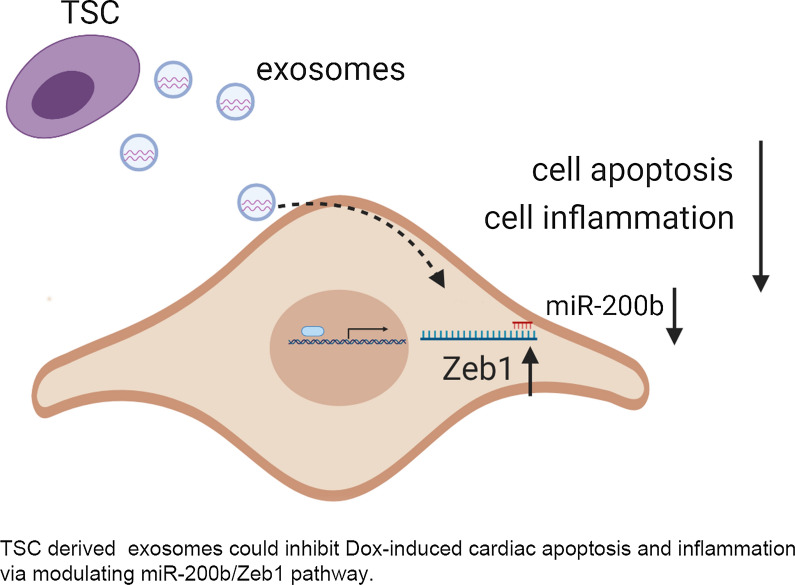

## Introduction

Cancer mortality has decreased during recent decades due to the widespread use of anthracyclines [1]. However, the number of patients suffering from the long-term effects of cardiotoxicity has increased [2, 3]. Several agents used in cancer treatment, such as doxorubicin (Dox), can adversely affect cardiac function and lead to congestive heart failure, which is a leading cause of disability in long-term survivors of cancer [4, 5] and a growing problem in the field of cardio-oncology.

Dox is an anthracycline cytostatic agent that has been in clinical use for almost a half century [6–8]. However, cumulative doses of Dox were found to be harmful to the myocardium [9], leading to left ventricular dysfunction and the development of heart failure, namely, Dox-induced cardiotoxicity. The underlying mechanisms include mitochondrial iron accumulation and reactive oxygen species (ROS) burst [10–13], eventually leading to cell apoptosis or cell necrosis [14–18]. However, the exact mechanisms have not been fully established, and optimal cardioprotective treatments remain undefined [8].

Various types of stem cells have been proposed for use in cardiac cell therapy [19, 20], such as skeletal myoblasts, mesenchymal stem cells (MSCs), hematopoietic stem cells (HSCs), and embryonic stem cells (ESCs). Trophoblast stem cells (TSCs) are a population of stem cells that originate from a single layer of blastocysts when the earliest cellular differentiation occurs [21]. Our previous results [22] illustrated that TSCs could improve cardiac function and attenuate myocardial adverse remodeling, which could be attributed to a lower level of miRNA-200b-3p. However, stem cell therapy is limited by an inconsistent supply, infusion toxicity, low survival and immune rejection. In addition, researchers confirmed that the cardioprotective effect of the administered stem cells was not derived from direct transdifferentiation into cardiomyocytes. Rather, this effect was mediated by the paracrine effects of exosomes secreted by stem cells [23, 24]. Therefore, in our study, we explored the effect of trophoblast cell-derived exosomes on Dox-induced cardiac injury and the underlying mechanism.

## Materials and methods

### Animal experiments

C57BL/6 mice (male, 8 weeks) were purchased from the Model Animal Research Center of Nanjing University. The animals were fed a standard laboratory diet with free access to food and water and housed in a temperature (22 ± 1°)- and humidity (65–70%)-controlled room with a 12-h light–dark cycle. After the study, all the animals were anesthetized by isoflurane inhalation (1.5–2%) and then euthanized by cervical dislocation. All the procedures with animals were approved by the Institutional Ethics Committee of Nanjing Drum Tower Hospital (Approval No. 20011141) and performed in accordance with the Care and Use of Laboratory Animals published by the National Institutes of Health (Eighth Edition).

Doxorubicin (Dox; Cat: D1515; Sigma; USA) was intraperitoneally administered to mice at a dose of 5 mg/kg once a week for 4 weeks (Dox groups). The Dox+Exo groups received an additional intramyocardial injection of 25 μl of PBS containing 50 μg TSC-Exos. The Dox+AAV groups received a tail vein injection of 25 µl (1*10^11 v.g.) of adeno-associated virus serotype 9 (AAV9; GeneChem; Shanghai), which carried miR-200b inhibitor (a small RNA to silence the expression of miR-200b), while the Dox+Vector groups received an equivalent dose of empty virus. All the groups were anesthetized by isoflurane inhalation (1.5–2%), ventilated with a rodent ventilator and subjected to thoracotomy for intramyocardial injection at 3 different points. Each group included 5 mice.

### Exosome isolation and identification

TSCs (HTR8-Svneo; Novobio; Shanghai) were cultured in DMEM containing 10% FBS and 1% penicillin/streptomycin (Gibco; USA) solution in 5% CO_2_ at 37 °C. When the cells reached 70 to 80% confluency, they were cultured with fresh DMEM containing 5% exosome-depleted fetal bovine serum (Cat: 161004-001; System Biosciences Inc; USA) and incubated for 48 h. Exosomes were isolated using differential centrifugation based on previously described methods with slight modifications [25, 26]. Briefly, the cells were harvested by centrifugation at 300×*g* for 10 min; the supernatant was then cleared of apoptotic bodies by centrifugation at 2000×*g* for 20 min; MVs were preferentially pelleted at 10,000×*g* for 30 min; and exosomes were then purified from the supernatant by ultracentrifugation at 100,000×*g* for 60 min. After isolation, 10 μl exosomes were diluted in 1 ml filtered PBS and stored at − 80 °C.

The morphology of the exosomes was observed using a transmission electron microscope (JEM1011; Japan), and the size distribution was measured using nanoparticle tracking analysis (NTA). For electron microscopy, we used 0.5% uranyl acetate to stain the exosomes and calculated their diameter from 5 to 15 images. For NTA, the samples were loaded into the sample chamber of an NS500 unit (Nanosight, Amesbury, UK), and five 1-min videos of each sample were recorded. Data analysis was performed with NTA 2.3 software (Nanosight), and the size and concentration of particles were calculated. Exosome markers (CD9, CD63 and TSG101) were detected by immunoblotting. To facilitate tracking in vitro, exosomes were labeled with the PKH67 Red Fluorescent Cell Linker Kit (Cat: PKH67GL; Sigma; USA).

### Cardiomyocyte culture and treatment

Primary cardiomyocytes were isolated from neonatal mice (post 1–2 days; Science of Chinese Academy of Medical Sciences; Beijing). In brief, hearts were enzymatically digested in HEPES-buffered saline solution containing 1.2 mg/ml pancreatin and 0.14 mg/ml collagenase at 37 °C. After centrifugation, the cells were collected and resuspended in DMEM. The dissociated cells were preplated at 37 °C for 1 h and then diluted to 1*10^6^ cells/ml and plated in 10 mg/ml laminin-coated culture dishes according to the specific experimental requirements. The Dox group received doxorubicin (1 µM) for 24 h. The Dox+Exo group received an additional 20 µg of exosomes. In addition, the cells were treated with miR-200b mimic (50 nM) (Dox+Mimic) and miR-200b inhibitor (50 nM) (Dox+Inhibitor), which were synthesized to overexpress miR-200b and inhibit miR-200b, respectively.

### Echocardiography

Cardiac function was assessed by transthoracic echocardiography (VEVO2100; visual sonics). The mice were anesthetized with isoflurane (1.5% in air) and monitored for respiratory frequency and temperature. End diastole was measured at the time of the apparent maximal LV diastolic dimension, and end systole was measured at the time of the most anterior systolic excursion of the posterior wall. The mice were assessed at 1 week before and 1, 2, 3 and 4 weeks after TSC-Exos injection. Left ventricular internal dimensions at end-diastole (LVIDd) and end-systole (LVIDs) were digitally measured on the M-mode tracings from 3 cardiac cycles. The left ventricular ejection fraction (EF) and left ventricular fractional shortening (FS) were calculated accordingly.

### RNA extraction and real-time RT-PCR

Total mRNA was extracted using a commercial kit (Cat: 9108Q; TaKaRa; Japan) according to the manufacturer’s instructions. Quantitative analyses were carried out on a real-time system (Applied Biosystems; USA) using SYBR Green PCR Master Mix (Cat: Q331-01; Vazyme, China) for mRNA or miRNA Universal SYBR qPCR Master Mix for miRNA (Cat: MQ101-01; Vazyme, China). The miRNA primers were obtained from Genescript (Nanjing). The relative expression level for each mRNA was calculated using the 2^−ΔΔCt^ method. The primers were as follows: ANP F: GCTTCCAGGCCATATTGGAG; R: GGGGGCATGACCTCATCTT; βMHC F: ACTGTCAACACTAAGAGGGTCA; R: TTGGATGATTTGATCTTCCAGGG; and Collagen I F: GCTCCTCTTAGGGGCCACT; R: CCACGTCTCACCATTGGGG.

### Western blotting

Heart tissues, exosomes and cells were lysed using RIPA buffer (Beyotime; Jiangsu). The protein concentration was measured by the BCA method (Thermo; USA). Equal amounts of protein were loaded on SDS-PAGE gels, separated and then transferred onto PVDF membranes (Millipore; USA). Then, the membranes were incubated with primary rabbit anti-mouse antibodies against CD9 (Cat: ab92726), CD63 (Cat: ab213090), TSG101 (Cat: ab125011), cleaved-caspase3 (Cat: ab2302), Bcl-2 (Cat: ab32124), Zeb1 (Cat: ab81972), p65 (Cat: ab16502) and GAPDH (Cat: ab181602) (Abcam; USA) at a dilution of 1:1000. After overnight incubation at 4 °C, the membranes were subsequently incubated with HRP-conjugated rabbit anti-mouse IgG (1:10000) for 1 h at room temperature. The immunobands were visualized using an enhanced chemiluminescence (ECL) detection kit (Cat: P0018S; Beyotime; China).

### Annexin V/PI staining for cell apoptosis

Primary cardiomyocytes were digested and collected at a concentration of 1*10^6 cells/ml. Then, the cells were resuspended in 200 µl binding buffer, and 5 µl Annexin-FITC and PI were added (Cat: KGA108-2; Keygen; China). After incubating for 30 min in the dark, the cells were analyzed by flow cytometry (BD; USA).

### Enzyme-linked immunosorbent assay (ELISA)

Murine blood was collected from the retro-orbital sinus and centrifuged at 3000 rpm for 5 min at 4 °C to acquire serum. The serum IL-1β (Cat: MLB00C) and IL-6 (Cat: M6000B) levels were measured using commercial enzyme-linked immunosorbent assay kits according to the manufacturer’s instructions (R&D; UK).

### Luciferase reporter measurement

The luciferase reporter clones with the 3′-untranslated region (UTR) of Zeb1 were purchased from GeneCopoeia (Rockville; MD). The clones included predicted miR-200b target sites acquired from an online database (www.mirdb.org) and a mutated sequence of the 3′UTR of Zeb1. The plasmids were transfected into HEK293 cells (200 ng per well) using Lipo 2000 (Life Technologies; USA) with 10 nM miR-200b mimic. After 24 h, the cell medium was collected for the luciferase assay.

### Histology assay

Heart tissues were harvested after 4 weeks. Then, the heart tissues were fixed in 4% paraformaldehyde (pH 7.4). After fixation and paraffin embedding, the cardiac tissues were cut into 5-μm-thick sections. The sections were stained with hematoxylin and eosin (Servicebio; China) to analyze the global heart morphology and inflammatory cell infiltration. The sections were stained with Masson’s trichrome (Servicebio; China) to evaluate cardiac fibrosis. The sections were scanned at 20-fold magnification on a high-resolution microscope (Leica; Japan).

### Statistical analysis

Data are presented as the mean ± standard derivation. All statistical analyses were performed with SPSS (23.0). Differences were analyzed with one-way analysis of variance (ANOVA) for multiple groups and Student’s t-test for only two groups. p < 0.05 was considered statistically significant.

## Results

### Characterization of TSC-derived exosomes

**Fig. 1:**
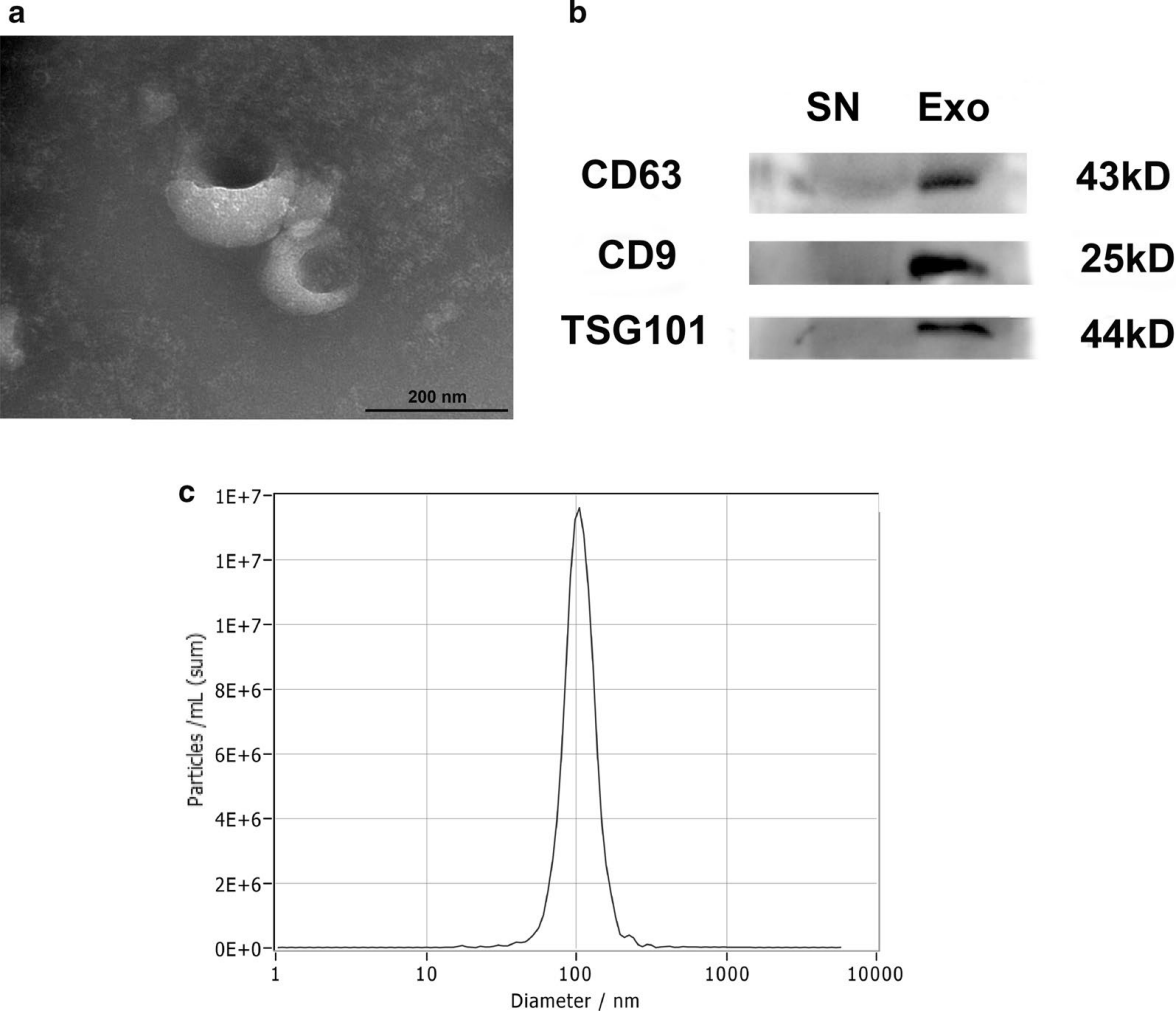
Characterization of TSC-derived exosomes. **a** The ultrastructure of exosomes was analyzed by transmission electron microscopy. **b** Western blot analysis showed the expression of protein markers of exosomes, such as CD63, CD9 and TSG101. **c** Representative DLS number distribution measurement of the isolated exosome population demonstrated an average diameter of 101 nm

Utilizing ultracentrifugation, we obtained exosomes from TSCs, as shown by TEM (Fig. 1a). The immunoblotting results (Fig. 1b) confirmed the expression of exosomal markers (CD63, CD9, and TSG101). In addition, DLS illustrated that the exosomes had an average diameter of 101 nm (Fig. 1c).

### TSC-derived exosomes protected cardiomyocytes from Dox-induced apoptosis

**Fig. 2:**
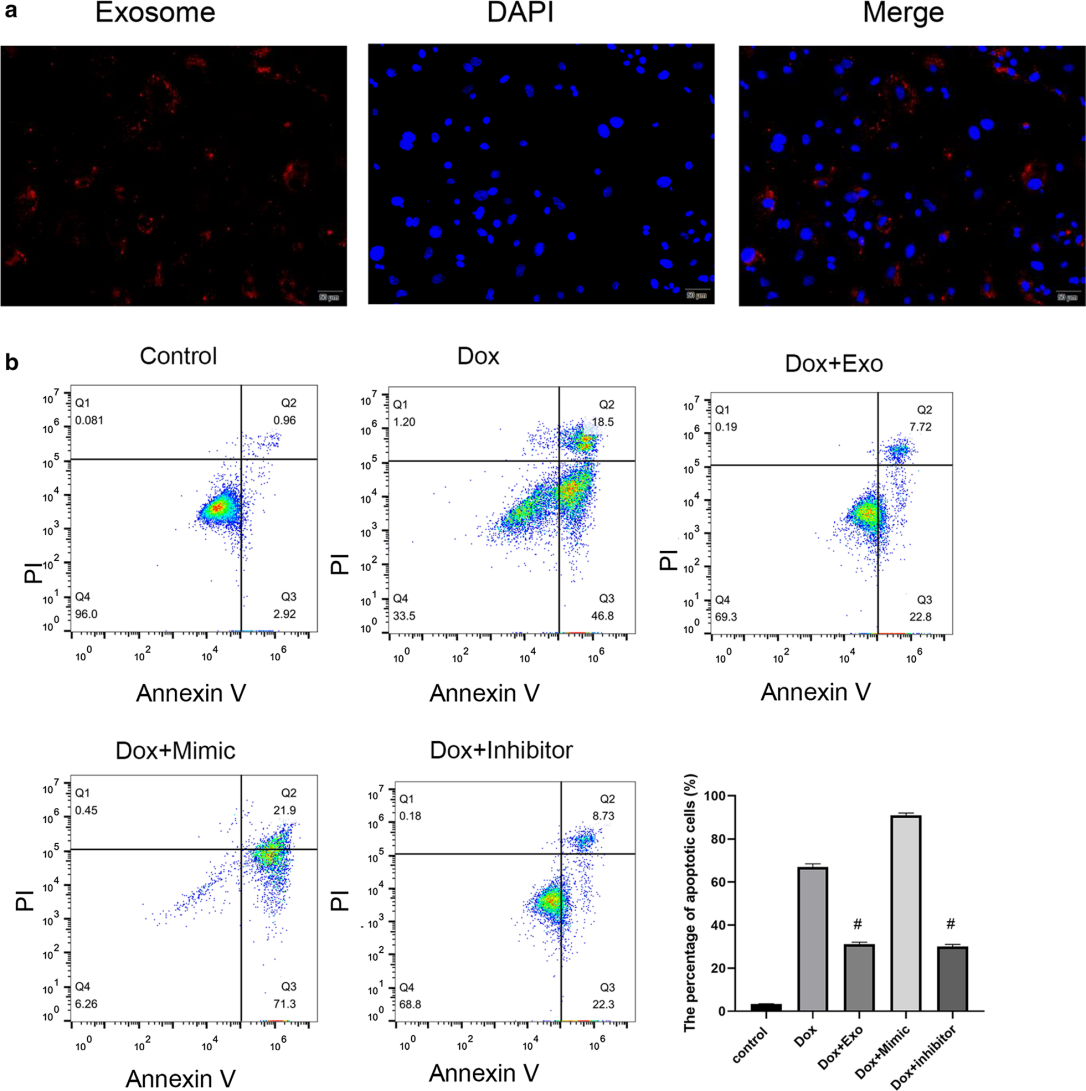
**a** The uptake of PKH67-labeled exosomes (red) by cardiomyocytes (blue). **b** The cardiomyocyte apoptosis level among groups is shown by flow cytometry. Dox indicates Dox-treated cardiomyocytes; Exo indicates Dox-treated cardiomyocytes incubated with TSC-Exos. Mimic indicates Dox-treated cardiomyocytes incubated with miR-200b mimic; Inhibitor indicates Dox-treated cardiomyocytes incubated with miR-200b inhibitor

**Fig. 3:**
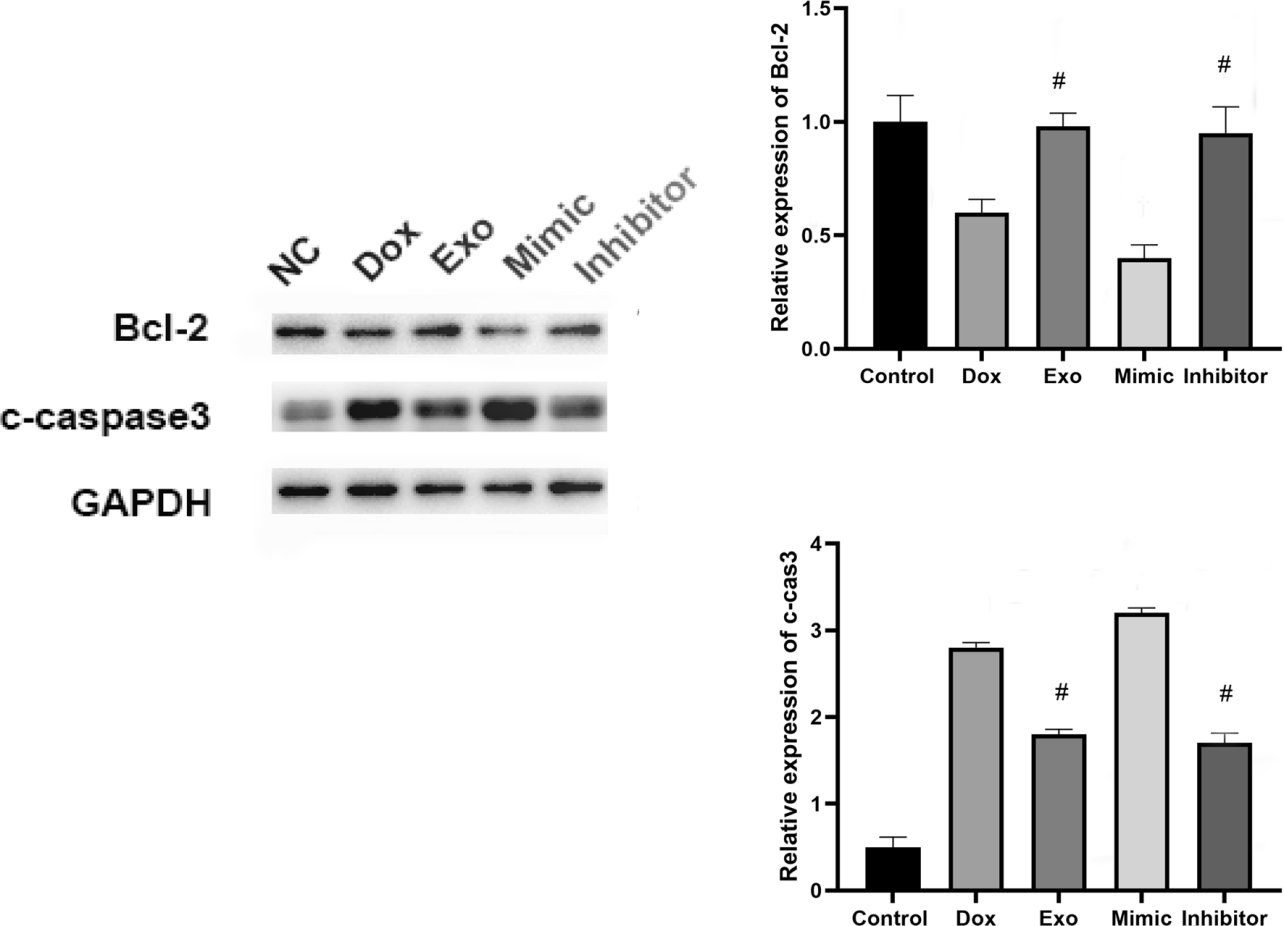
**a** TSC-Exos exerted antiapoptotic effects on Dox-treated cardiomyocytes. The expression of Bcl-2 and caspase3 by western blotting (left) and quantitative analysis (right). Dox indicates Dox-treated cardiomyocytes; Exo indicates Dox-treated cardiomyocytes incubated with TSC-Exos; Mimic indicates Dox-treated cardiomyocytes incubated with miR-200b mimic; Inhibitor indicates Dox-treated cardiomyocytes incubated with miR-200b inhibitor. #p < 0.05 compared with Dox

As shown in Fig. 2a, labeled exosomes were taken up by primary cardiomyocytes. Previous studies showed that TSCs could decrease the expression of miR-200b in cardiac tissues [22]. Therefore, we treated primary cardiomyocytes with TSC-Exos as well as miR-200b mimic and inhibitor. The flow cytometry results (Fig. 2b) showed that exosomes and inhibitors both inhibited cell apoptosis. In addition, exosome- and inhibitor-treated cardiomyocytes had lower C-caspase 3 expression and higher Bcl-2 expression than the cardiomyocytes in other groups (Fig. 3).

### TSC-Exos improved cardiac function in Dox-induced heart failure

**Fig. 4:**
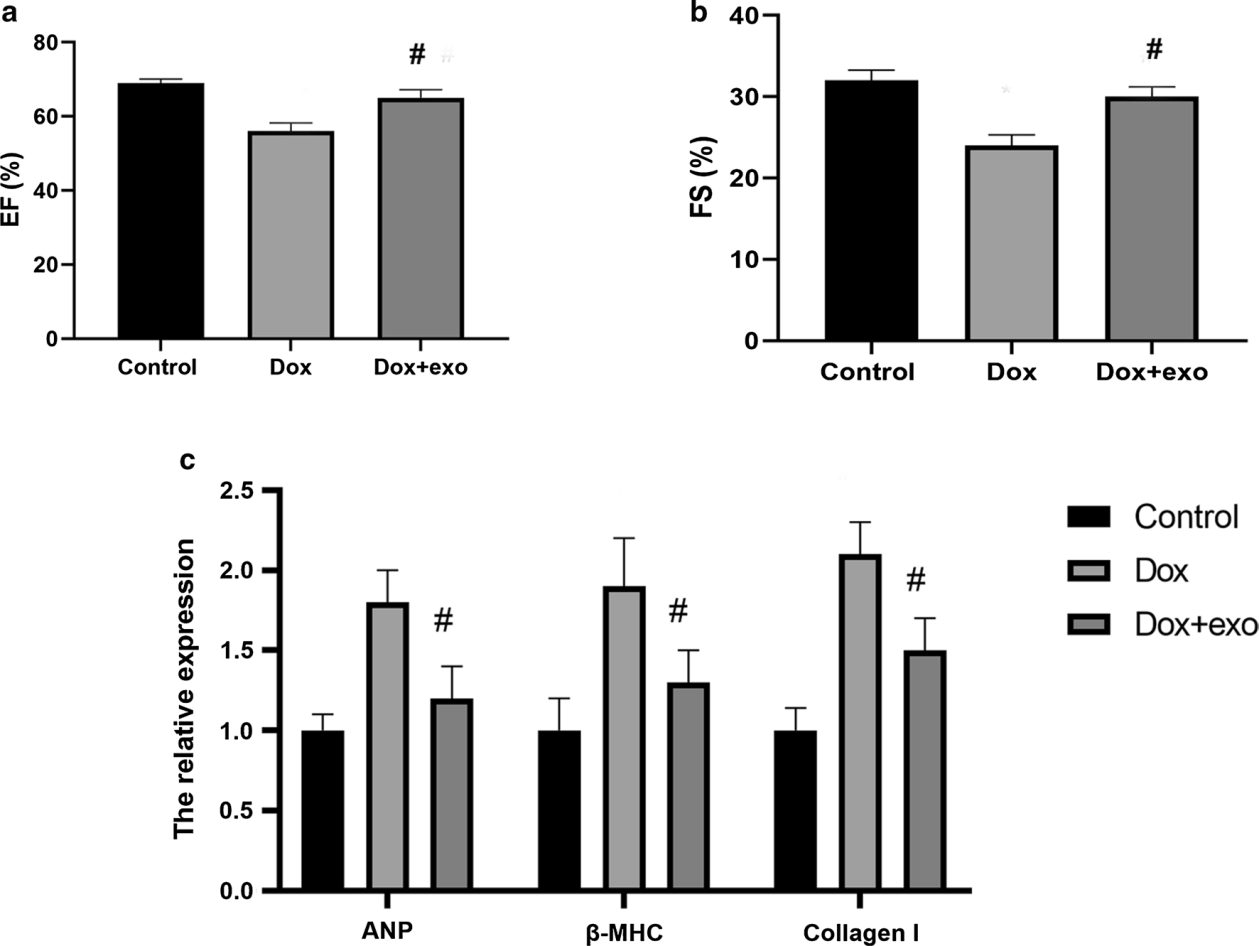
Echocardiographic data of different groups after 4 weeks. **a** Left ventricular ejection fraction (EF); **b** left ventricular fraction shortening (FS); **c** mRNA expression of heart failure markers, such as ANP, MHC and Collagen I. Dox indicates Dox-treated mice injected with PBS; Dox+Exo indicates Dox-treated mice injected with TSC-Exos. #p < 0.05 compared with Dox

Dox-treated mice were treated with or without exosomes to determine the effect of exosomes in vivo. After 4 weeks, the echocardiographic results (Additional file 1: Table S1) showed that exosome-treated mice had a higher EF and FS (Fig. 4a, b), suggesting a favorable role of the exosomes in cardiac remodeling. Additionally, heart failure-associated markers (ANP, β-MHC, and Collagen I) showed decreased expression in the exosome group (Fig. 4c).

### TSC-Exos decreased miR-200b expression and played a protective role in vivo

**Fig. 5:**
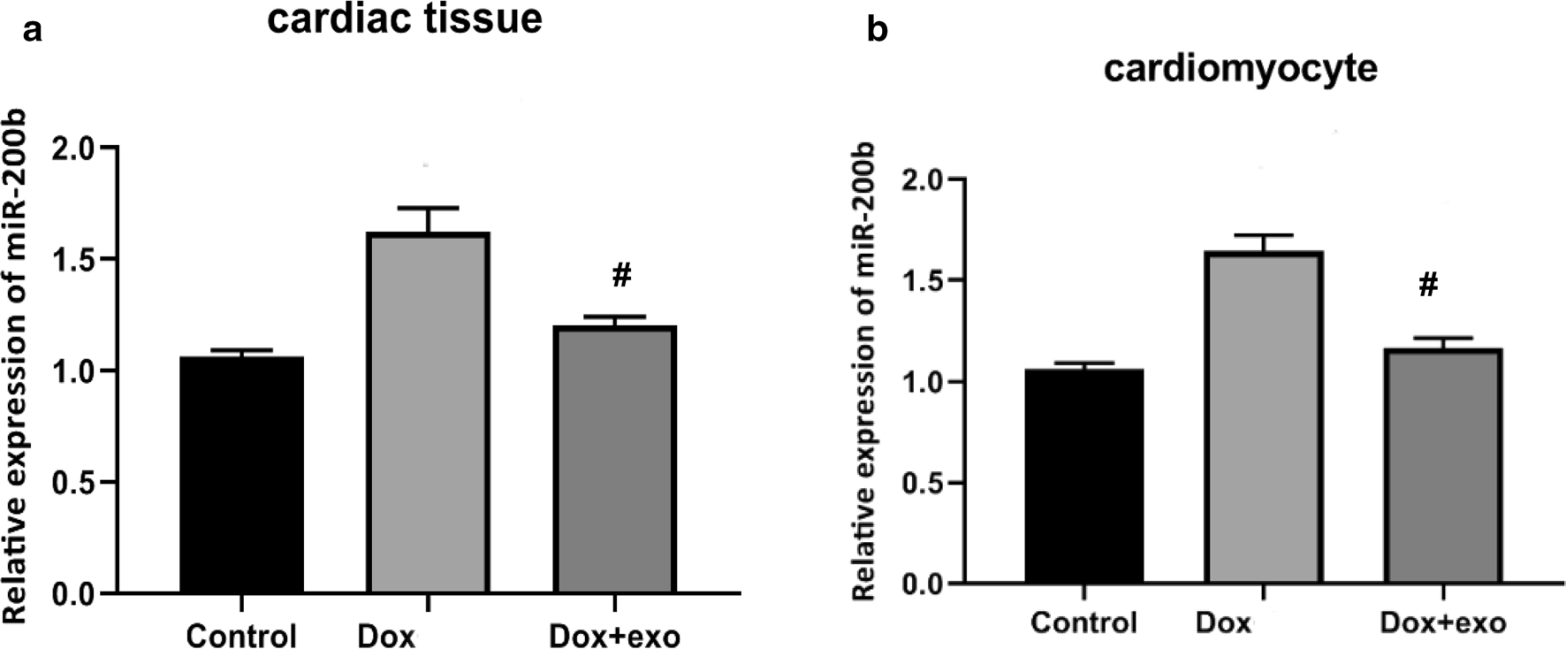
**a** The expression of miR-200b in heart tissues of Dox-treated mice; **b** the expression of miR-200b in Dox-treated cardiomyocytes. Dox indicates Dox-treated mice injected with PBS; Dox+Exo indicates Dox-treated mice injected with TSC-Exos. #p < 0.05 compared with Dox

**Fig. 6:**
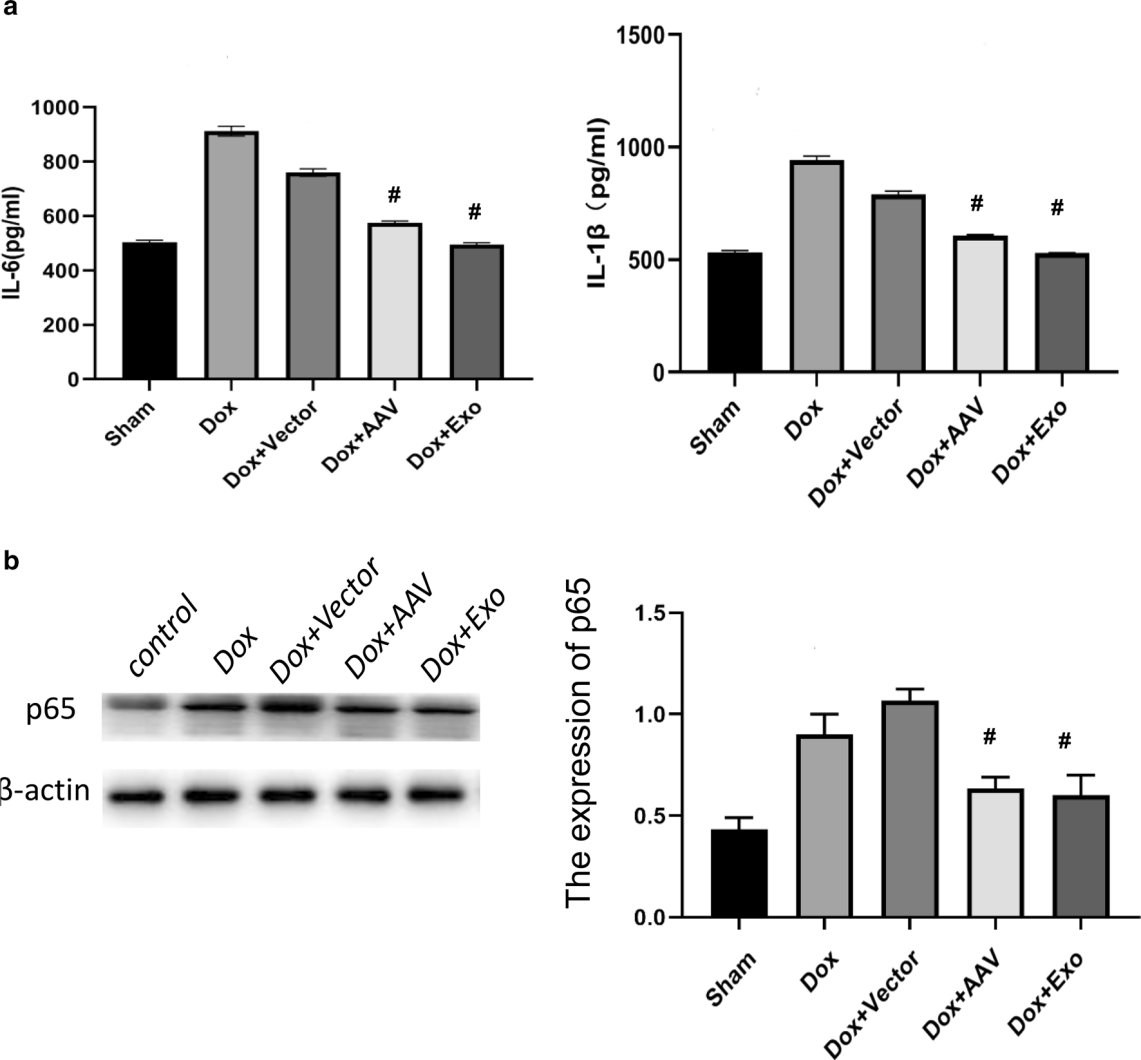
TSC-Exos exert anti-inflammatory effects in Dox-induced cardiac injury. **a** ELISA analysis of the concentration of inflammatory factors, including IL-1β and IL-6; **b** the expression of p65 by western blotting. Dox+Vector indicates Dox-treated mice injected with AAV9 vector; Dox+AAV indicates Dox-treated mice injected with AAV9-miR-200b inhibitor; Dox+Exo indicates Dox-treated mice injected with TSC-Exos. #p < 0.05 compared with the Dox+Vector group

**Fig. 7:**
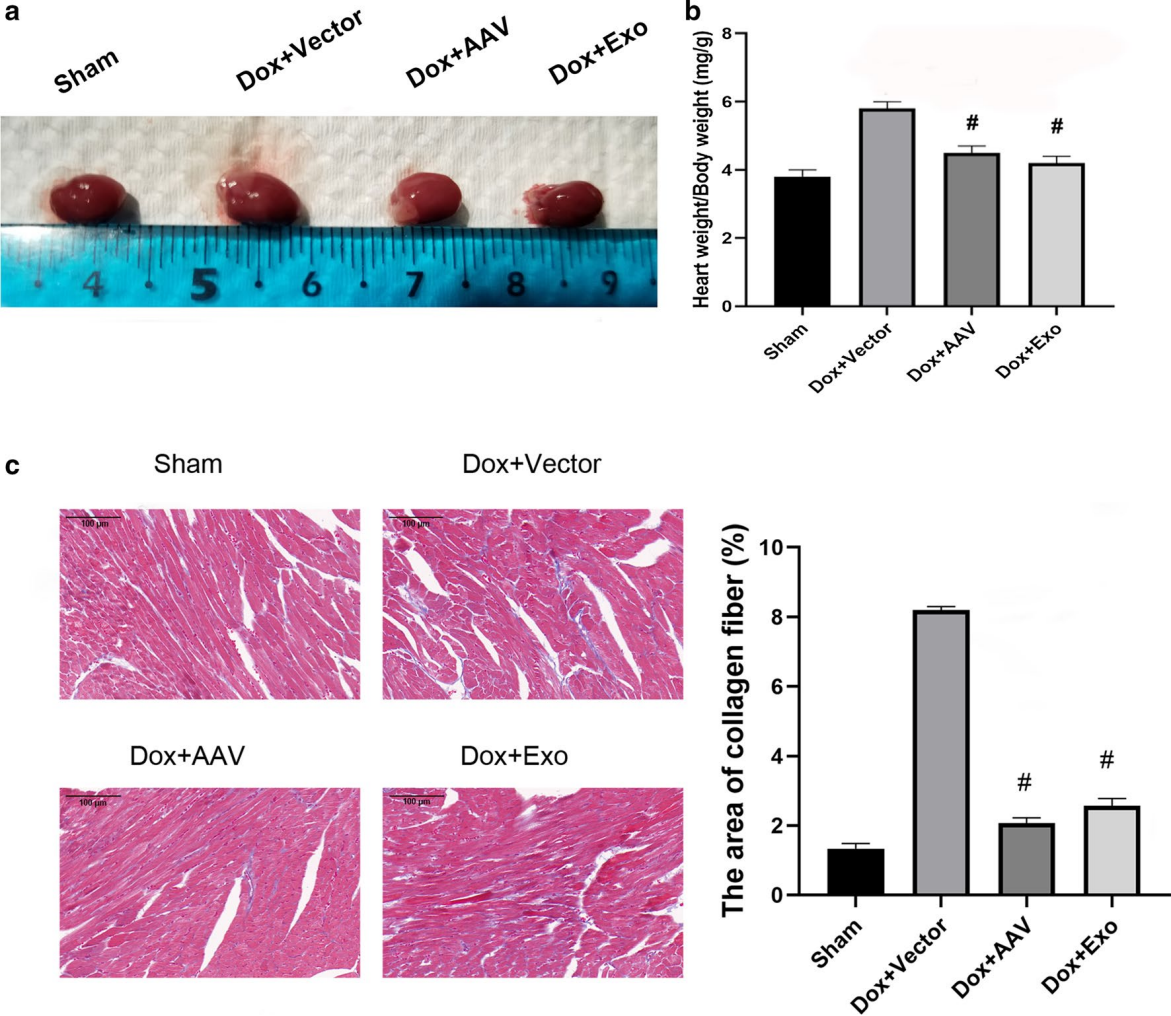
**a** The morphology of the hearts of different groups; **b** the heart weight to body weight ratio of different groups; **c** the fibrotic areas of different groups by Masson staining. Right is ×20 magnification of the corresponding area labeled with a black rectangle in the left whole LV ring. Dox+Vector indicates Dox-treated mice injected with AAV9 vector; Dox+AAV indicates Dox-treated mice injected with AAV9-miR-200b inhibitor; Dox+Exo indicates Dox-treated mice injected with TSC-Exos. #p < 0.05 compared with the Dox+Vector group

miR-200b was downregulated in both TSC-Exo-treated cardiac tissues and primary cardiomyocytes (Fig. 5a, b), suggesting that miR-200b is involved in the protective role of TSC-Exos. To further confirm the role of miR-200b, miR-200b was inhibited by transfecting the miR-200b inhibitor AAV. The exosome group and inhibitor group exhibited decreased levels of serum IL-1β and IL-6 (Fig. 6a) and cardiac p65 (Fig. 6b), suggesting decreased systemic inflammation and local inflammation. Moreover, exosomes and miR-200b inhibitor both attenuated the heart size (Fig. 7a), the heart weight to body weight ratio (Fig. 7b), and cardiac fibrosis (Fig. 7c), suggesting a favorable role of these treatments in cardiac remodeling.

### Zeb1 was a target of miR-200b involved in the protective role of TSC exosomes

**Fig. 8:**
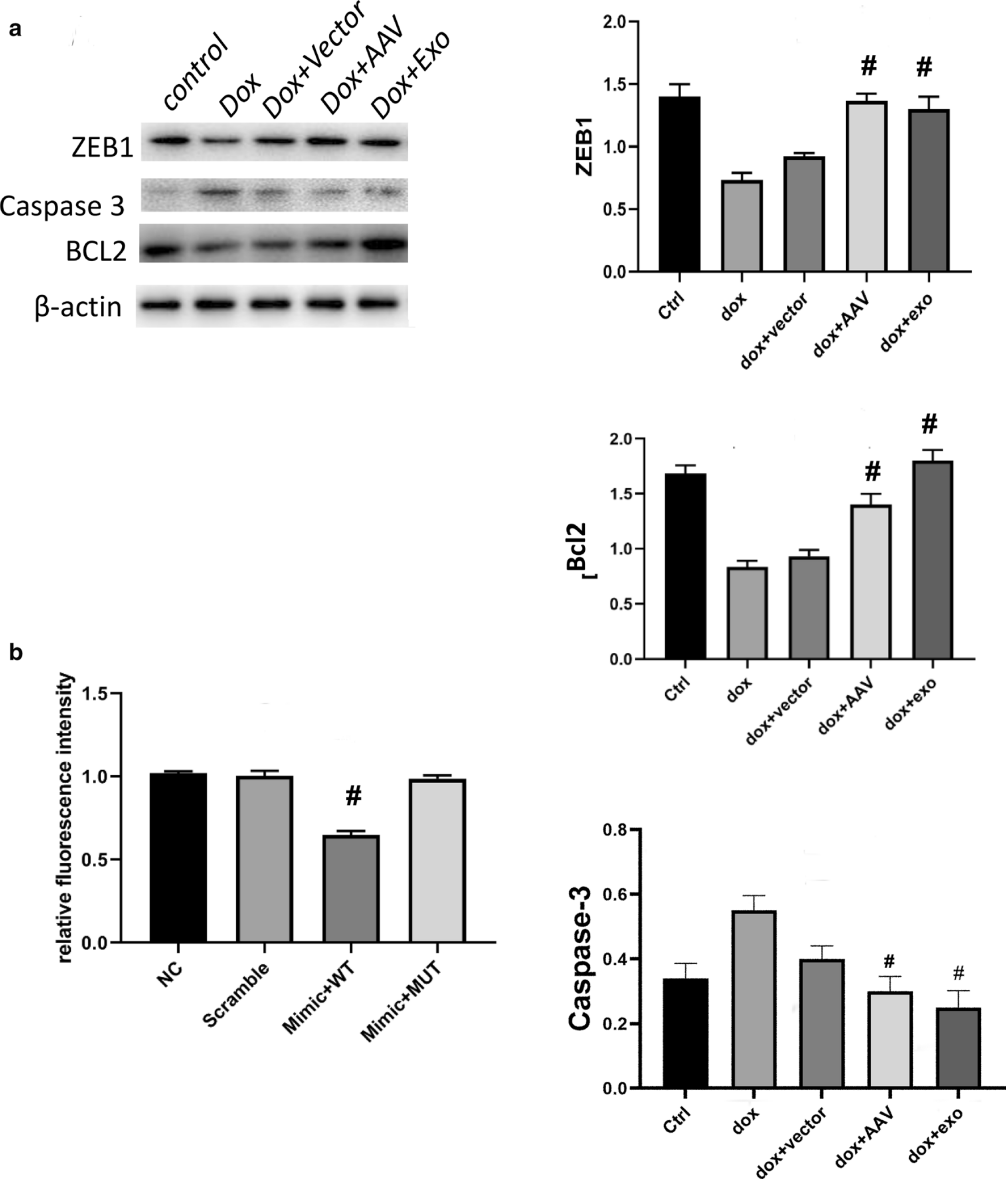
TSC-Exos exert anti-apoptotic effects on tissues with Dox-induced cardiac injury. **a** The expression of Zeb1, Caspase3 and Bcl-2 was measured by western blotting, and β-actin was used as the reference control; the quantitative results are shown on the right. Dox indicates Dox-treated mice; Dox+Vector indicates Dox-treated mice injected with AAV9 vector; Dox+AAV indicates Dox-treated mice injected with AAV9-miR-200b inhibitor; Dox+Exo indicates Dox-treated mice injected with TSC-Exos. **b** The luciferase results showing miR-200b binding to Zeb1. Scramble indicates cells transfected with the scramble sequence; Mimic+WT indicates cells transfected with the miR-200b-3p and wild type 3′UTR of Zeb1; Mimic+MUT indicates cells transfected with the miR-200b-3p and mutated 3′UTR of Zeb1. #p < 0.05 compared with Dox or Scramble

Previous studies revealed that Zeb1 was a downstream target of miR-200b and exerted an antiapoptotic effect. Therefore, we examined Zeb1 expression in the abovementioned groups. In addition to decreased apoptosis, the exosome and overexpression groups both exhibited increased Zeb1 expression (Fig. 8a). The luciferase reporter assay also confirmed the direct binding of miR-200b to Zeb1 (Fig. 8b).

## Discussion

Our studies revealed, for the first time, that TSC-Exos can protect murine hearts from Dox-induced injury by regulating miR-200b expression. Specifically, TSC-Exos exerted an antiapoptotic and anti-inflammatory effect by decreasing the expression of Zeb1 and some inflammatory cytokines, which provided an alternative mechanism of reversing chemotherapy-induced heart failure.

MicroRNAs (miRNAs), as a class of small noncoding RNAs [27], have emerged as posttranscriptional regulators that function by binding to the 3′-UTR of target genes [28]. miRNAs are involved in cell proliferation, differentiation, metastasis, apoptosis, and immune responses [29]. Doxorubicin could cause cardiac injury by inducing the expression of some miRNAs. For example, a study confirmed a strong association of miR-34a-5p and miR-451a with Dox-induced cardiotoxicity [30]. miR-21 suppression or miR-130a prevented cardiac dysfunction induced by doxorubicin [31, 32]. Altered miRNAs could be a potential biomarker or early toxicity signature, which was reviewed in some publications [33, 34]. In our study, the expression of miR-200b was upregulated in Dox-treated cardiomyocytes and was negatively correlated with the expression of Bcl2, indicating that miR-200b was a proapoptotic mediator. Our results showed that TSC-Exos decreased miR-200b expression in cardiomyocytes and inhibited apoptosis in cardiomyocytes by upregulating the anti-apoptotic Bcl-2 protein. miR-200 members were reported to promote cell apoptosis in cancer [26, 35], which was consistent with our result that miR-200 mimic aggravated cardiac injury suffering from Dox injection. Korpal et al. had previously reported that miR-200b could target Zeb1 [36]. A luciferase reporter was constructed to confirm the direct binding of miR-200b and Zeb1. However, it remains unclear how TSC-Exos regulate the expression of miR-200b. Some studies reported that TSC-Exos could play a role by delivering noncoding RNAs, proteins and cytokines to recipient cells [37–39]. We hypothesized that lncRNAs enriched in TSC-Exos could transfer to hearts and bind to and silence miR-200b via a ceRNA-dependent mechanism, which needs to be confirmed by future experiments.

Recent findings illustrated that Dox activated the NF-κB pathway to cause inflammatory effects on the myocardium and vasculature [40]. Conversely, blocking the NF-κB pathway in cardiac microvascular endothelial cells could weaken inflammatory damage [41]. Our data showed that IL-1b, IL-6 and p65 were increased in the Dox and miR-200b mimic groups but decreased in the exosome and inhibitor groups. In addition, we monitored echocardiography in animal models and found that cardiac function was significantly improved in animal models treated with exosomes. These results suggested that exosomes could inhibit the inflammatory response, thereby protecting cardiomyocytes and improving cardiac function subjected to Dox injury.

## Conclusion

In summary, our study elucidated the antiapoptotic role of TSC-Exos in injured cardiomyocytes, which was achieved by inhibiting miR-200b while increasing Zeb1 expression. This study provides a new strategy to treat heart failure in the future.

## Supplementary information

**Additional file 1: Table S1.** The echocardiographic parameters of the control group, Dox group, Dox+Exo group, Dox+Vector group and Dox+AAV group (n=5 each group).

## Data Availability

All the data can be obtained upon reasonable request.

## Abbreviations

TSC: Trophoblast stem cells
TSC-Exos: Trophoblast stem cell-derived exosomes
Dox: Doxorubicin
AAV: Adeno-associated virus
ROS: Reactive oxygen species
MSCs: Mesenchymal stem cells
HSCs: Hematopoietic stem cells
ESCs: Embryonic stem cells
LVIDd: Left ventricular internal dimensions at end-diastole
LVIDs: Left ventricular internal dimensions at end-systole
EF: Ejection fraction
FS: Fractional shortening
ELISA: Enzyme-linked immunosorbent assay
UTR: 3′-Untranslated region
ANOVA: One-way analysis of variance
