# Supplementary material for: Human trophoblast-derived exosomes attenuate doxorubicin-induced cardiac injury by regulating miR-200b and downstream Zeb1
Source: J Nanobiotechnology. 2020 Nov 20;18:171. doi: 10.1186/s12951-020-00733-z (PMC7678329; doi:10.1186/s12951-020-00733-z)
Supplement: Supplementary file 1 — Additional file 1: Table S1. The echocardiographic parameters of the control group, Dox group, Dox+Exo group, Dox+Vector group and Dox+AAV group (n=5 each group). [file 12951_2020_733_MOESM1_ESM.docx]

Table S1. The echocardiographic parameters of the control group, Dox group, Dox+Exo group, Dox+Vector group and Dox+AAV group (n=5 each group).

| Parameters | Control | Dox | Dox+Exos | Dox+Vector | Dox+AAV |
| --- | --- | --- | --- | --- | --- |
| LVID; d, mm | 3.87±0.08 | 4.07±0.06 | 3.99±0.10 | 4.10±0.08 | 3.98±0.07 |
| LVID; s, mm | 2.63±0.05 | 3.08±0.07 | 2.80±0.05 | 3.05±0.10 | 2.83±0.08 |
| LVPW; d, mm | 0.64±0.05 | 0.62±0.08 | 0.63±0.01 | 0.61±0.05 | 0.64±0.10 |
| LVPW; s, mm | 0.98±0.02 | 0.80±0.05 | 0.91±0.06 | 0.82±0.08 | 0.90±0.07 |
| EF, % | 69±1.1 | 56±2.2 | 65±2.2^#^ | 55±2.0 | 66±2.3^*^ |
| FS, % | 32±1.3 | 24±1.3 | 30±1.2^#^ | 25.6±1.1 | 28.9±1.0^*^ |

Abbreviations: LVID; d, left ventricular internal end-diastolic diameter; LVID; s, left ventricular internal end-systolic diameter; LVPW; d, left ventricular post-wall end-diastolic diameter; LVPW; s, left ventricular post-wall end-systolic diameter; EF, left ventricular ejection fraction; FS, left ventricular fractional shortening.

*p<0.05 compared with the Dox+Vector group;

#P<0.05 compared with the Dox group.
